# Shenqi Fuzheng injection facilitates skeletal muscle mitophagy mediated by the ubiquitination of HIF‐1α to ameliorate cancer‐associated fatigue

**DOI:** 10.1111/jcmm.18455

**Published:** 2024-06-19

**Authors:** Wei Guo, Shan Liu, Huan Xia, Jiamin Luo, Hanrui Chen, Leihao Hu, Xinting Zheng, Zhiwei Xiao, Lizhu Lin

**Affiliations:** ^1^ Department of Oncology The First Affiliated Hospital of Guangzhou University of Chinese Medicine Guangzhou Guangdong China; ^2^ Lingnan Medical Research Center Guangzhou University of Chinese Medicine Guangzhou Guangdong China; ^3^ Science and Technology Innovation Center Guangzhou university of Chinese Medicine Guangzhou China; ^4^ Geriatrics Research Institute Sichuan Provincial People's Hospital Chengdu Sichuan China

**Keywords:** cancer‐related fatigue, HIF‐1α, mitophagy, PINK1, Shenqi Fuzheng injection

## Abstract

Cancer‐related fatigue (CRF) significantly impacts the quality of life of cancer patients. This study investigates the therapeutic potential of Shenqi Fuzheng injection (SFI) in managing CRF, focusing on its mechanistic action in skeletal muscle. We utilized a CRF mouse model to examine the effects of SFI on physical endurance, monitoring activity levels, swimming times and rest periods. Proteomic analysis of the gastrocnemius muscle was performed using isobaric tags and liquid chromatography–tandem mass spectrometry to map the muscle proteome changes post‐SFI treatment. Mitochondrial function in skeletal muscle was assessed via ATP bioluminescence assay. Furthermore, the regulatory role of the hypoxia inducible factor 1 subunit alpha (HIF‐1α) signalling pathway in mediating SFI's effects was explored through western blotting. In CRF‐induced C2C12 myoblasts, we evaluated cell viability (CCK‐8 assay), apoptosis (flow cytometry) and mitophagy (electron microscopy). The study also employed pulldown, luciferase and chromatin immunoprecipitation assays to elucidate the molecular mechanisms underlying SFI's action, particularly focusing on the transcriptional regulation of PINK1 through HIF‐1α binding at the PINK1 promoter region. Our findings reveal that SFI enhances physical mobility, reduces fatigue symptoms and exerts protective effects on skeletal muscles by mitigating mitochondrial damage and augmenting antioxidative responses. SFI promotes cell viability and induces mitophagy while decreasing apoptosis, primarily through the modulation of HIF‐1α, PINK1 and p62 proteins. These results underscore SFI's efficacy in enhancing mitochondrial autophagy, thereby offering a promising approach for ameliorating CRF. The study not only provides insight into SFI's potential therapeutic mechanisms but also establishes a foundation for further exploration of SFI interventions in CRF management.

## 1 INTRODUCTION

Cancer‐related fatigue (CRF) is a symptom of cachexia that is prevalent across all stages of tumour development.^1^, ^2^, ^3^ Unlike general fatigue, CRF persists regardless of rest or sleep, impacting 30% to 60% of individuals with moderate or severe CRF. Even after effective treatment, long‐term CRF may be experienced in 25% to 33% of cases, even for over a decade.^4^, ^5^ The unrelenting nature of CRF significantly deteriorates cancer patients' quality of life, posing a substantial clinical challenge. Several interventions, encompassing exercise programs, psychosomatic therapies and psychoeducation, have been developed to alleviate CRF.^6^, ^7^, ^8^ However, a standardized treatment protocol remains elusive. Furthermore, CRF often leads patients with cancer to discontinue subsequent treatments, severely impeding their prognosis. Consequently, finding effective interventions to alleviate CRF is imperative for enhancing the outcomes of patients with cancer.

The aetiology of CRF is intricate and multifaceted, with the underlying mechanisms remaining largely unknown. Current research posits that CRF emerges from a confluence of factors, including the tumour itself, treatment‐related complications and sociopsychological influences.^9^, ^10^ Chemotherapy and radiotherapy, while targeting tumours, can also adversely affect nontumor tissues, including nervous tissue and skeletal muscle.^3^ The resulting toxicity disrupts mitochondrial structure and function in skeletal muscles, reducing the energy supply to muscle cells and elevating oxidative stress, thereby precipitating CRF.^11^, ^12^, ^13^ Consequently, investigating skeletal muscle mitochondrial dysfunction has emerged as a pivotal area of research, enhancing our comprehension of CRF pathogenesis.

Shenqi Fuzheng injection (SFI), an injected traditional Chinese medication primarily consisting of ginseng and astragalus, has garnered attention due to its anticancer properties.^14^, ^15^ Clinical applications of SFI as an adjuvant treatment for lung and gastric cancers have exhibited promising results. Notably, SFI has been linked to enhanced chemotherapy efficacy when combined with treatments for colorectal and breast cancers, concurrently bolstering patients' immune functions.^16^, ^17^ Studies have hinted at SFI's potential to counteract immunosuppression and promote the cytotoxic activity of immune cells in the tumour microenvironment.^18^, ^19^ Preliminary investigations have also shown that SFI can alleviate depressive symptoms in mouse cancer models, reduce tumour growth and reduce fatigue through the inhibition of inflammatory factors secreted by immune cells.^19^, ^20^ Moreover, SFI reduces exhaustion in T cells and promotes antitumor immune responses. However, the precise mechanism underlying the impact of SFI on CRF remains elusive.

The influence of skeletal muscle on CRF and the pivotal role of mitochondrial dysfunction in this context form a crucial area of research in understanding and treating CRF. Skeletal muscles, beyond their mechanical functions, are integral to whole‐body metabolism and energy homeostasis. In the setting of cancer, the catabolic state induced by the tumour and its treatments leads to skeletal muscle wasting, known as cachexia, which is closely associated with CRF. This muscle wasting is not merely a consequence of reduced physical activity but is driven by a complex interplay of inflammatory cytokines, neuroendocrine factors and other tumour‐derived factors. These agents not only reduce muscle mass but impair muscle function, contributing significantly to the fatigue experienced by cancer patients.

Mitochondrial dysfunction within skeletal muscles plays a critical role in the pathogenesis of CRF. Mitochondria are the powerhouse of cells, responsible for producing the majority of the cell's supply of adenosine triphosphate (ATP), used as a source of chemical energy. In cancer, alterations in mitochondrial structure and function are observed, including reduced mitochondrial biogenesis, changes in mitochondrial morphology and impaired oxidative phosphorylation. These changes lead to a decreased ATP production and increased production of reactive oxygen species, further exacerbating muscle fatigue. The resulting energy deficit and oxidative stress contribute to the sensation of persistent fatigue that is not relieved by rest, characteristic of CRF.

SFI, a traditional Chinese medicinal formulation consisting mainly of ginseng and astragalus, has been explored for its potential in mitigating CRF through its multifaceted actions on immune modulation, inflammation reduction and possibly mitochondrial protection. SFI is posited to enhance the body's resilience against the tumour‐induced catabolic state, partly by improving immune function and reducing the secretion of pro‐inflammatory cytokines that contribute to muscle wasting and mitochondrial dysfunction. Moreover, SFI's potential effects on promoting mitochondrial biogenesis and improving mitochondrial function could play a significant role in alleviating CRF by restoring energy balance and reducing oxidative stress in skeletal muscles. While the precise mechanisms of SFI's actions on mitochondrial function in the context of CRF remain to be fully elucidated, existing evidence points to its promise as a complementary treatment strategy aimed at improving the quality of life for cancer patients suffering from CRF.

In an effort to unravel this complexity, we devised a comprehensive CRF model in mice, simulating tumour‐induced fatigue‐like behaviour. Subsequently, we administered SFI to ascertain its potential in alleviating fatigue symptoms. Concurrently, we meticulously examined inflammation‐ and immune‐related indicators to discern the influence of SFI on the body's immunity. Employing a multifaceted approach, our study integrated network pharmacology with animal and cellular experiments. These endeavours were undertaken with the explicit objective of comprehensively exploring the effect of SFI on CRF and elucidating its underlying mechanisms. Through this rigorous methodology, we aimed to provide a foundation for novel interventions for CRF to improve the overall well‐being and clinical outcomes of cancer patients.

## 2 MATERIALS AND METHODS

A detailed description was provided in the Supplementary materials—Data S1.

### 2.1 Statistical analysis

All data were derived from carefully conducted experiments spanning three distinct iterations, ensuring robust and reliable results. These data are presented as the mean values, accompanied by their corresponding standard errors of the mean, ensuring accuracy and reliability. To rigorously evaluate the differences among various experimental groups, a comprehensive one‐way analysis of variance (ANOVA) was employed for statistical analysis. All statistical assessments were conducted utilizing GraphPad Prism 7 software, a powerful tool renowned for its precision and reliability in data analysis. The significance levels were set at *p* < 0.05 and *p* < 0.01, representing stringent criteria for identifying statistical significance amid the observed differences.

## 3 RESULTS

### 3.1 Influence of SFI on weight and fatigue in CRF model mice

**FIGURE 1:**
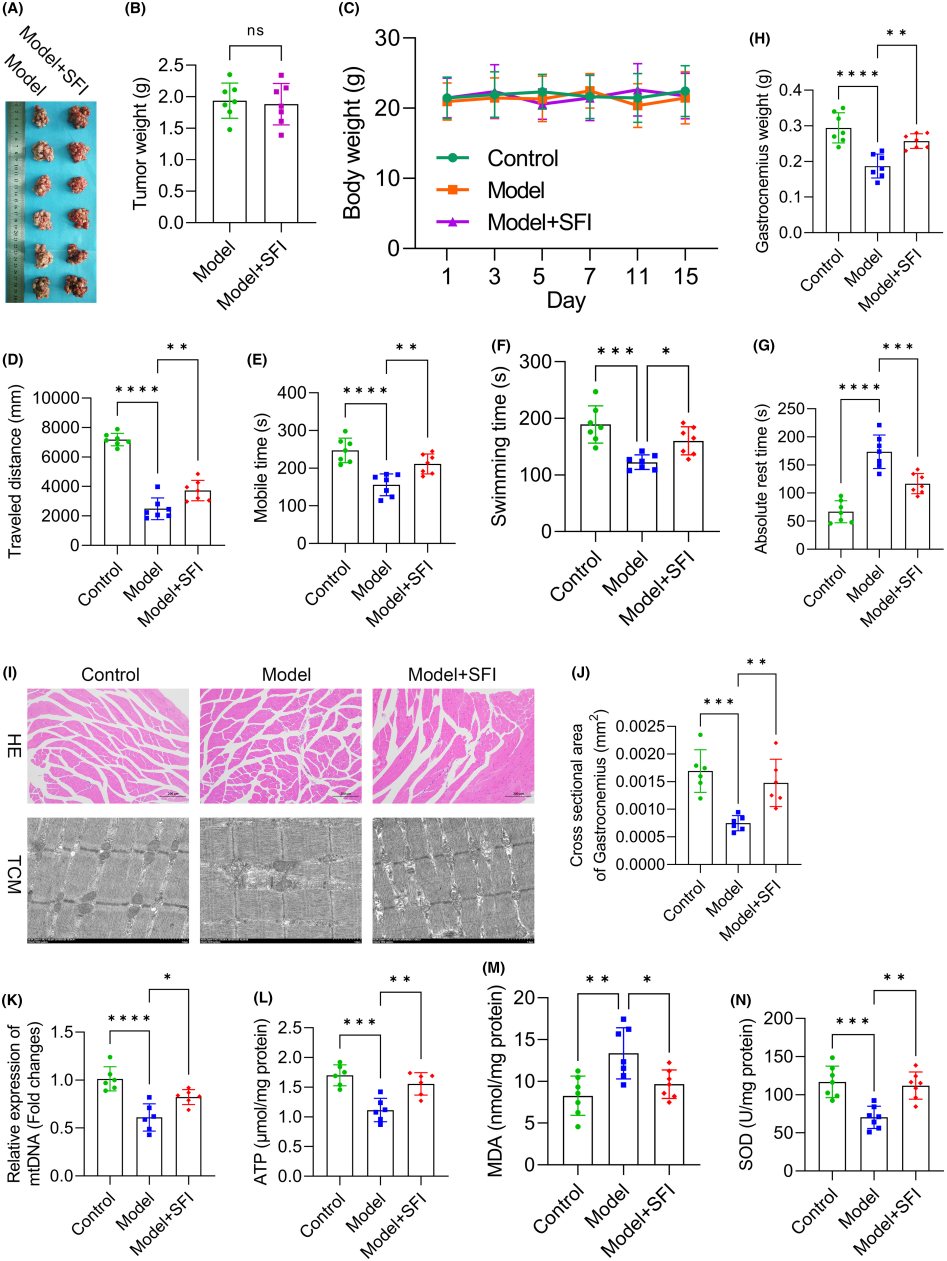
Shenqi Fuzheng injection (SFI) enhances mitochondrial function and alleviates oxidative stress as well as gastrocnemius weight reduction in cancer‐related fatigue (CRF) mouse models. Images of the tumour samples taken from mice after 28 days (A). Effects of SFI on tumour weights (B) and body weights (C). Effects of SFI on distance moved (D), duration of mobility (E), duration of swimming (F), duration of rest (G) and gastrocnemius weight (H) in CRF model mice. HE‐stained sections and electron micrographs showing mitochondrial structures (I) (Scale bar = 200 μm). Cross‐sectional areas of gastrocnemius muscle after SFI treatment (J) (Scale bar = 1 μm) and relative levels of mtDNA (K), adenosine triphosphate (ATP) (L), malondialdehyde (MDA) (M) and superoxide dismutase (SOD) (N) in CRF model mice. ****p < 0.0001, ***p < 0.001; **p < 0.01; *p < 0.05. ns, nonsignificant.

In our quest to decipher the roots of CRF and evaluate the therapeutic efficacy of SFI, we analysed mouse samples. Figure 1A shows images of the tumours postsampling, with comparable weights observed between the SFI and model groups (*p* > 0.050; Figure 1B). Body weight fluctuations were minor across 15 days postinjection for all groups (Figure 1C). Our open‐field test results showed reductions in distances moved (*p* < 0.0001; Figure 1D) and duration of movement (*p* < 0.0001; Figure 1E) in the model group relative to the control group, confirming successful CRF model establishment. Interestingly, the SFI group displayed increased distances (*p* < 0.010; Figure 1D) and durations of mobility (*p* < 0.010; Figure 1E) compared to the model group. The exhaustive swimming test indicated reduced swimming time in the model mice (vs. the control group; *p* < 0.001) but a notable increase in SFI‐treated mice (vs. the model group; *p* < 0.050; Figure 1F). The tail suspension test demonstrated prolonged rest in the model mice (vs. the control group; *p* < 0.0001) and reduced rest time in the SFI‐treated mice (vs. the model group; *p* < 0.001; Figure 1G). These results underscore SFI's potential in mitigating CRF symptoms.

### 3.2 Effects of SFI on oxidative stress and mitochondrial function in skeletal muscles in CRF model mice

Our investigations revealed significant changes in the gastrocnemius, shedding light on the impact of SFI. The model group displayed reduced gastrocnemius weight (vs. the control group; *p* < 0.0001), whereas the SFI group showed an increase (vs. the model group; *p* < 0.010; Figure 1H), highlighting SFI's potential in combating myophagism. HE staining indicated the ability of SFI to mitigate skeletal muscle cell damage (Figure 1I). Electron microscopy imaging further revealed that CR induced structural disarray and looseness in muscle fibres, contrasting the order observed in the control group (Figure 1I). Gastrocnemius cross‐sectional areas were diminished in model mice (vs. the control group; *p* < 0.001) but were increased in SFI‐treated mice (vs. the model group; *p* < 0.010; Figure 1,J), underscoring SFI's muscle‐preserving effects. Notably, mitochondrial assessments revealed substantial differences. CRF led to diminished mitochondrial DNA (mtDNA) (*p* < 0.0001, Figure 1K), adenosine triphosphate (ATP) (*p* < 0.001, Figure 1L) and superoxide dismutase (SOD) levels (*p* < 0.001, Figure 1N), while malondialdehyde (MDA) levels increased (*p* < 0.010, Figure 1M) compared to the control group. In contrast, the SFI group exhibited elevated mtDNA, ATP and SOD levels (mtDNA: *p* < 0.050; ATP and SOD: both *p* < 0.010), coupled with decreased MDA levels (*p* < 0.050), indicating SFI's capacity to enhance muscle mitochondrial function and mitigate oxidative stress. These findings illuminate SFI's potential for preserving muscle integrity and function in CRF.

### 3.3 Effects of SFI on serum biochemical indices in CRF model mice

**FIGURE 2:**
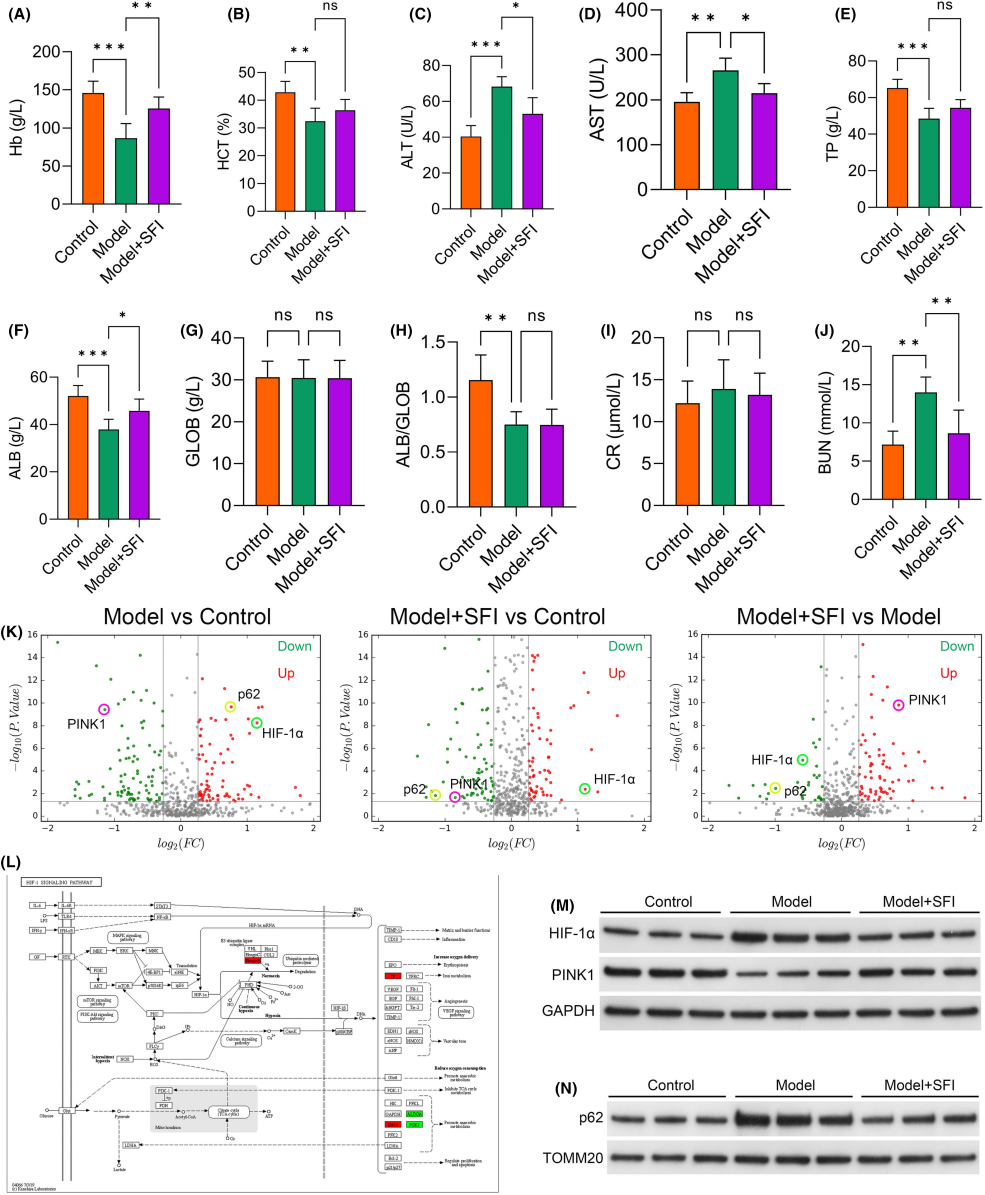
Shenqi Fuzheng injection (SFI) may alleviate hepatic function impairment via the HIF‐1α pathway in cancer‐related fatigue (CRF) model mice. Effects of SFI on Hb (A), hematocrit (HCT) (B), alanine aminotransferase (ALT) (C), aspartate aminotransferase (AST) (D), total protein (TP) (E), albumin (ALB) (F), globulin (GLOB) (G), ALB/GLOB (H), creatinine (CR) (I) and blood urea nitrogen (BUN) (J). Volcano plot illustrating the differentially expressed proteins (DEPs) between the control, model and SFI groups. Proteins with the highest differential expression are shown at the top, with upregulated proteins coloured red and downregulated proteins coloured green; grey colour indicates no significant change. (K). Proteomic profiling of gastrocnemius muscles using isobaric tags for relative and absolute quantification (iTRAQ) and LC–MS/MS. Differentially expressed proteins (DEPs) associated with CRF were identified based on a 1.2‐fold increase or a 0.83‐fold decrease in abundance and *p* < 0.05. (L) The DEPs to KEGG enrichment analysis and confirmed that SFI potentially regulates the HIF‐1α pathway. (M, N) The protein expression levels of HIF‐1α, PINK1 and p62 were assessed via western blot analysis. HIF‐1α and p62 exhibited increased levels in the model mice (vs. the control group) but was downregulated in the SFI‐treated mice. Conversely, PINK1 was downregulated in model mice (vs. the control group) but was increased in the SFI‐treated group compared with model group. ***p < 0.001; **p < 0.01; *p < 0.05; ns, nonsignificant.

In the subsequent assessment of hepatic impairment in the CRF mouse model, we observed the impact of SFI treatment. Haemoglobin (Hb) levels (*p* < 0.001; Figure 2A) and haematocrit (HCT) values (*p* < 0.010; Figure 2B) were decreased in the model group relative to the control group. Notably, Hb levels were markedly increased in the SFI‐treated group relative to the model mice (*p* < 0.010; Figure 2A), indicating SFI's potential to mitigate cancer‐induced anaemia. Additionally, HCT exhibited an increasing trend (though not statistically significant) in the SFI group relative to the model mice (*p* > 0.050; Figure 2B). The liver enzyme markers alanine aminotransferase (*p* < 0.001; Figure 2C) and aspartate aminotransferase (*p* < 0.010; Figure 2D) were elevated in the model group relative to the control group but were markedly reduced in the SFI‐treated mice (*p* < 0.050 for both). Total protein (*p* < 0.001), albumin (ALB) (*p* < 0.001) and the ALB/Globulin (GLOB) ratio (*p* < 0.010) were lower in the model mice than in the control group, while GLOB levels showed no significant change (*p* > 0.050; Figure 2E–H). Among these, only ALB was elevated in the SFI mice relative to the model group (*p* < 0.050), while TP, GLOB and ALB/GLOB exhibited no significant differences (all *p* > 0.050). Furthermore, though increases in CR (creatinine) were observed in the model mice, these increases were not statistically significant (vs. the control group; *p* > 0.050), and blood urea nitrogen (BUN) levels were increased in the model mice relative to the control group (*p* < 0.010). However, CR levels were not different between the SFI and model groups (*p* > 0.050; Figure 2I), and BUN was significantly reduced in the SFI group compared with the model group (*p* < 0.010; Figure 2J). These results underscore SFI's potential in alleviating impaired liver function, although its effect on mitigating renal damage was comparatively moderate.

### 3.4 SFI activated HIF‐1α in CRF model mice

Subsequently, we conducted an in‐depth analysis of the gastrocnemius proteome in CRF mice using isobaric tags for relative and absolute quantification (iTRAQ) and LC–MS/MS. Following stringent criteria (1.2‐fold increase or 0.83‐fold decrease, *p* < 0.05), 191 proteins were identified as differentially expressed proteins (DEPs), including 103 downregulated and 88 upregulated proteins in model group compared with control group, 185 proteins were identified as DEPs, including 105 downregulated and 83 upregulated proteins in SFI intervention group compared with control group, and 129 proteins were identified as DEPs, including 42 downregulated and 87 upregulated proteins in SFI intervention group compared with model group (Figure 2K). The protein quantification results were then visually depicted in a volcano plot. Notably, p62 and Hypoxia inducible factor 1 subunit alpha (HIF‐1α) exhibited increased levels in model mice relative to the control group, while PINK1 was downregulated. Remarkably, the SFI group exhibited significant upregulation of PINK1 and substantial reductions in HIF‐1α and p62 compared to the model group (Figure 2K). To gain deeper insights into the molecular pathways and networks underlying CRF, we subjected the DEPs to KEGG enrichment analysis and confirmed that SFI potentially regulates the HIF‐1α pathway (Figure 2L). To verify these findings, the protein expression levels of HIF‐1α, PINK1 and p62 were assessed via western blot analysis (Figure 2M,N). HIF‐1α and p62 exhibited increased levels in the model mice (vs. the control group) but was downregulated in the SFI‐treated mice. Conversely, PINK1 was downregulated in model mice (vs. the control group) but was increased in the SFI‐treated group compared with model group. These observations suggested that HIF‐1α may be the primary pathway modulated by SFI. Based on these comprehensive analyses, the HIF‐1α pathway was selected for subsequent analyses.

### 3.5 Effects of SFI on cell viability, apoptosis and oxidative stress in C2C12 myotubes

**FIGURE 3:**
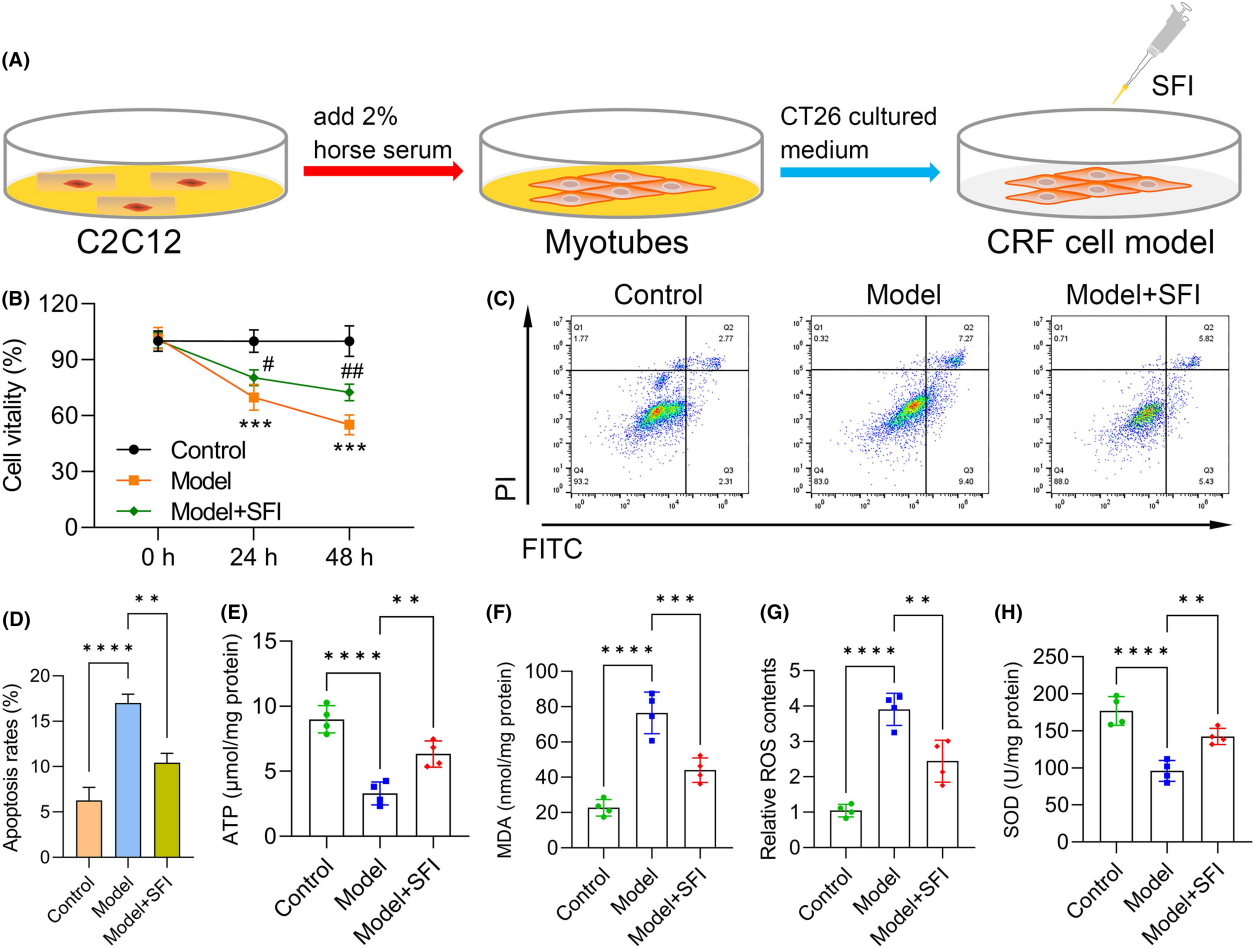
Shenqi Fuzheng injection (SFI) promotes cell viability and reduces apoptosis and oxidative stress in C2C12 myotubes. Flowchart of the cancer‐related fatigue (CRF) cell model induction procedure (A). Effects of SFI on C2C12 cell viability (C) and apoptosis (D) in CRF cell models. AV‐PI staining showing the effects of SFI on apoptosis (C). Effects of SFI on the levels of adenosine triphosphate (ATP) (E), malondialdehyde (MDA) (F), reactive oxygen species (ROS) (G) and superoxide dismutase (SOD) (H). ****p < 0.0001, ***p < 0.001; **p < 0.01. ^##^ *p* < 0.01; ^##^ *p* < 0.05.

We next performed CRF cell model induction as previously described, with the flowchart of the C2C12 induction procedure shown in Figure 3A. C2C12 cell viability was significantly reduced in the model group (vs. the control group), while this phenomenon was reversed to some extent in SFI‐treated mice (vs. the model group) (Figure 3B). Apoptosis was increased in the model group (vs. the control group; *p* < 0.0001) and reduced after treatment with SFI (vs. the model group; *p* < 0.010) (Figure 3C,D). Furthermore, we observed that ATP was significantly inhibited in the model group (*p* < 0.0001) but was elevated in the SFI‐treated group (vs. the model group; *p* < 0.010; Figure 3E), suggesting that SFI enhanced mitochondrial function in C2C12 cells. Levels of MDA (Figure 3F) and ROS (Figure 3G) were increased in the model group (vs. the control group; both *p* < 0.0001), while they were reduced in the SFI‐treated group (vs. the model group; MDA: *p* < 0.001; ROS: *p* < 0.010). Conversely, SOD was downregulated in the model group (vs. the control group; *p* < 0.0001), but it was upregulated in the SFI‐treated group (vs. the model group; *p* < 0.010; Figure 3H), indicating that SFI reduced oxidative stress levels in gastrocnemius muscles.

### 3.6 Influence of SFI on C2C12 cell ultrastructures and mitochondrial autophagosomes following culture in CT26‐conditioned medium

**FIGURE 4:**
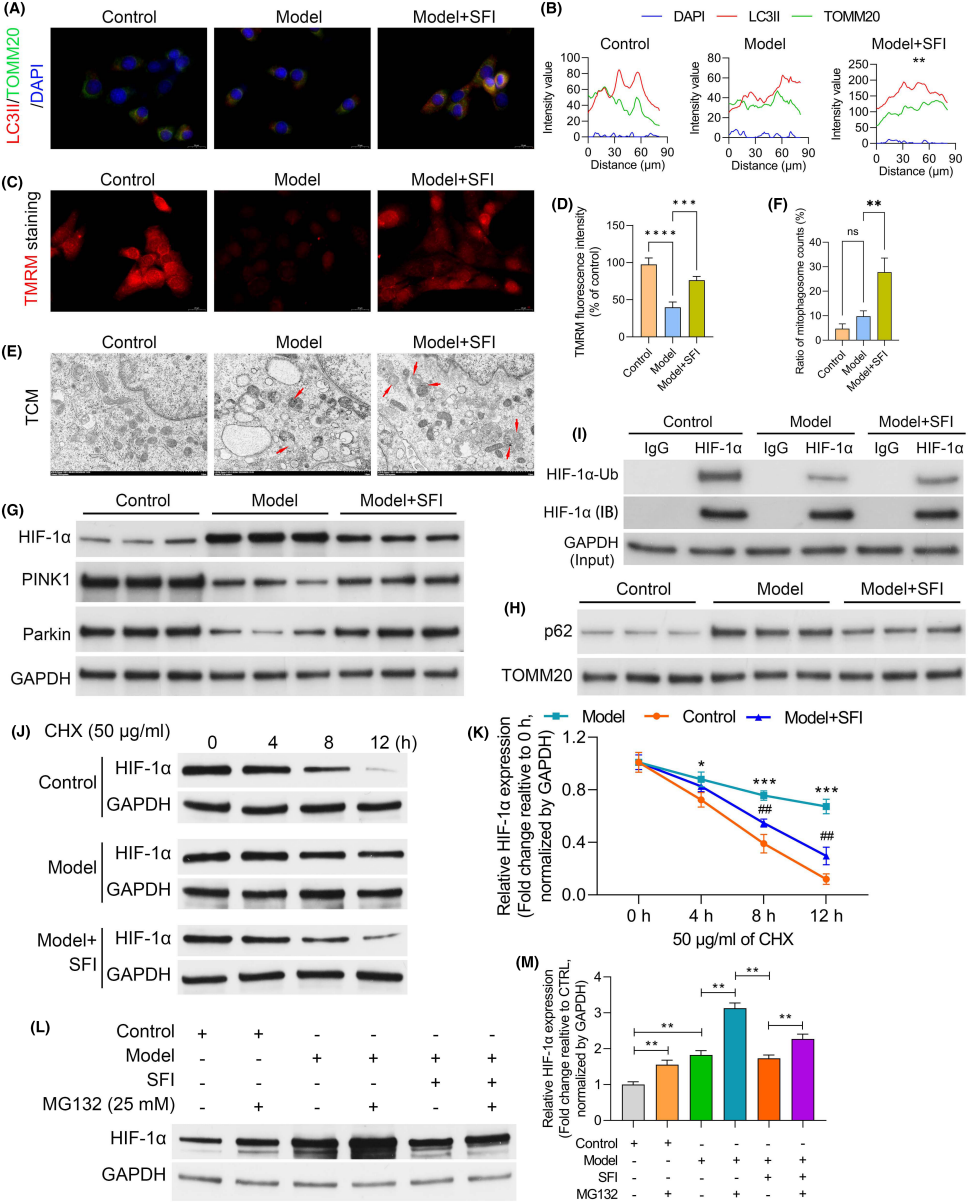
Effects of Shenqi Fuzheng injection (SFI) on C2C12 cell ultrastructure and mitochondrial autophagosomes after culture with CT26‐conditioned medium. (A) Double immunofluorescence staining of LC3‐II and TOMM20 (Scale bar = 20 μm). (B) The model group significantly reduced the formation of LC3‐positive autophagosomes colocalized with mitochondria, while SFI reversed this phenomenon. (C, D) Effect of SFI on mitochondrial membrane potential measured by tetramethyl rhodamine methyl ester perchlorate (TMRM) uptake (Scale bar = 20 μm). (E, F) Electron micrographs of cells in each group and the results of the statistical analysis of the mitochondrial fragments (Scale bar = 1 μm). (G) Western blots showing the protein levels of PTEN induced kinase 1 (PINK1) and Parkin. (H) Western blots showing the protein levels of p62. (I) Analysis of hypoxia inducible factor 1 subunit alpha (HIF‐1α) ubiquitination and degradation. (J) Immunoblot analysis of HIF‐1α levels in whole‐cell extracts. (K) Densitometric analysis of the HIF‐1α protein bands, whereby the relative fold change in the level is with respect to the level at 0 h. (L) C2C12 cells treated with SFI and MG132 (25 mM) for 12 h; immunoblot analysis of HIF‐1α levels in the C2C12 cells. (M) Densitometric analysis of the HIF‐1α protein bands, whereby the relative fold change in the level is with respect to the level with control. ****p < 0.0001; ***p < 0.001; **p < 0.01. ns, nonsignificant.

In our immunofluorescence analysis employing microtubule associated protein 1 light chain 3 (LC3)‐II and translocase of outer mitochondrial membrane 20 (TOMM20) costaining, a notable decrease in LC3‐positive autophagosomes colocalized with mitochondria was observed in the model mice relative to the control group. This reduction was effectively reversed by SFI treatment (Figure 4A,B). Subsequent evaluation of mitochondrial membrane potential using tetramethyl rhodamine methyl ester perchlorate (TMRM) uptake revealed a marked decrease in membrane potential in C2C12 cells treated with CT26‐conditioned medium relative to the control group. However, this reduction was reversed in the presence of SFI (Figure 4C,D). Additionally, as illustrated in Figure 4E,F, stimulation with CT26‐conditioned medium led to a nonsignificant elevation in the proportion of cells showing mitochondrial fragmentation compared with the control group. However, SFI treatment reduced both mitochondrial fragmentation and autophagosome numbers. Further analysis of the HIF‐1α pathway indicated marked upregulation of HIF‐1α protein levels in the CT26‐conditioned medium group relative to the control group. However, this increase was markedly attenuated in the SFI group (Figure 4G). Mitochondrial morphology is intricately linked to mitochondrial function. Therefore, assessments of key functional markers, including PINK1, Parkin and p62, were performed in the control, CT26‐conditioned medium and CT26‐conditioned medium + SFI groups using GAPDH and TOMM20 as internal references. In the CT26‐conditioned medium group, the levels of both PINK1 and Parkin were significantly lower than those in the control group, while SFI treatment elevated their expression levels (Figure 4G). Conversely, the p62 levels were notably higher in the CT26‐conditioned medium model group, and SFI slightly mitigated this effect (Figure 4H). Subsequent ubiquitination analysis revealed significant ubiquitination‐mediated degradation of HIF‐1α in model mice relative to the control group. However, SFI intervention effectively inhibited this degradation process (Figure 4I). Furthermore, considering the effect of SFI on the HIF‐1α protein level, we treated C2C12 cell with the protein synthesis inhibitor cycloheximide and found that the protein stability and half‐life of HIF‐1α were dramatically decreased after SFI treatment in C2C12 cell (Figure 4J,K). In addition, HIF‐1α protein degradation in C2C12 cells was decelerated after SFI treatment and increased by treatment with the proteasome inhibitor MG132 (Figure 4L,M), suggesting that SFI promoted proteasome‐dependent degradation of HIF‐1α in C2C12 cell. The above results implying that SFI has positive effects on the ultrastructure and mitochondrial autophagosomes of C2C12 cells cultured in CT26‐conditioned medium.

### 3.7 Dependence of mitophagy on cellular HIF‐1α expression

**FIGURE 5:**
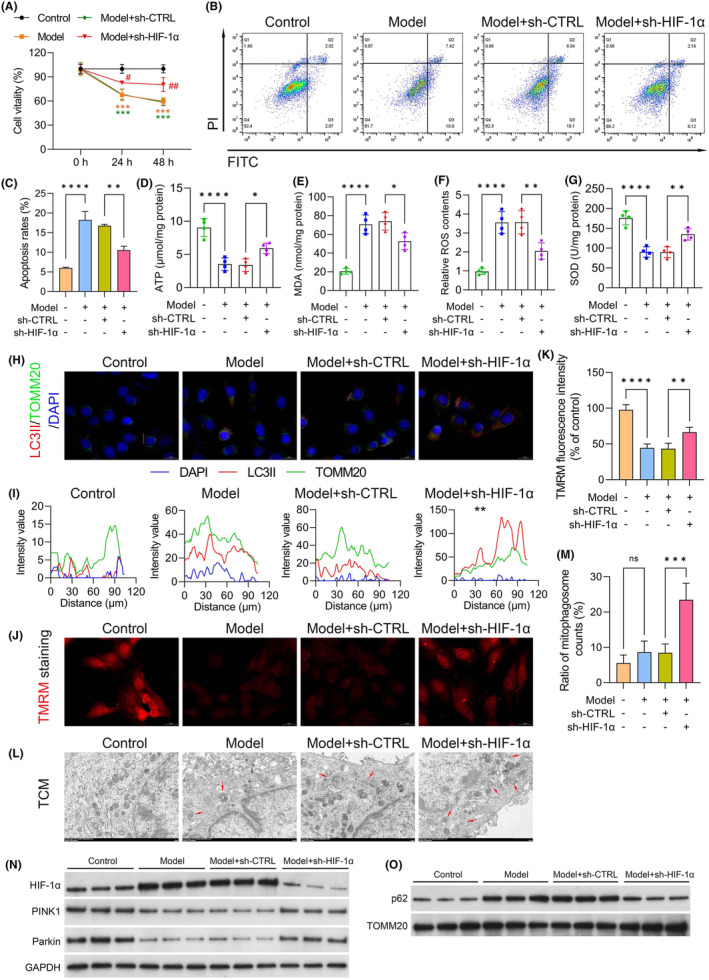
Dependence of mitophagy on cellular hypoxia inducible factor 1 subunit alpha (HIF‐1α) expression. (A) CCK‐8 assay of cell viability. (B, C) Flow cytometry evaluation of apoptosis. (D, E) Levels of adenosine triphosphate (ATP) and malondialdehyde (MDA). (F, G) Levels of reactive oxygen species (ROS) and superoxide dismutase (SOD). (H) Lysosome identification using anti‐microtubule associated protein 1 light chain 3 (LC3) II and translocase of outer mitochondrial membrane 20 (TOMM20) antibodies (Scale bar = 20 μm). (I) Colocalization analysis of LC3II and TOMM20. (J, K) TMRM uptake assessment of mitochondrial membrane potential in the cancer‐related fatigue (CRF) model (Scale bar = 20 μm). (L, M) Electron micrographs of cells showing changes in the number and appearance of mitochondria (Scale bar = 1 μm). (N, O) Western blots showing the levels of proteins in the PTEN induced kinase 1 (PINK1)–Parkin pathway. ****p < 0.0001; ***p < 0.001; **p < 0.01; *p < 0.05; ns, nonsignificant.

To investigate the impact of HIF‐1α on mitophagy, shRNA was used to silence HIF‐1α. In the Model + sh‐CTRL group, C2C12 cell viability was markedly lower than that in the cells in the control group (*p* < 0.001). However, this reduction in cell viability was reversed in the Model + sh‐HIF‐1α group (*p* < 0.050; Figure 5A). Apoptosis analysis indicated significant enhancement of apoptosis in cells cultured with CT26‐conditioned medium (model group) and in the model group transfected with sh‐CTRL compared with the control group. Conversely, apoptosis was significantly inhibited following HIF‐1α silencing, as evidenced by Annexin V‐FITC/PI staining (Figure 5B,C). Moreover, we observed reductions in ATP (Figure 5D) and SOD (Figure 5G) levels in the model (vs. the control group; both *p* < 0.0001) and Model + sh‐CTRL groups, which were partially restored upon transfection with sh‐HIF‐1α. These results suggest that SFI might enhance mitochondrial function by downregulating HIF‐1α in the CRF cell model. Conversely, the MDA (Figure 5E) and ROS (Figure 5F) levels were increased in the model (vs. the control group; both *p* < 0.0001) and Model + sh‐CTRL groups but were significantly reduced in the Model + sh‐HIF‐1α groups. These results indicate that downregulation of HIF‐1α alleviated oxidative stress in the CRF cell model. Immunostaining was used for the assessment of mitophagy by staining cells with LC3II and TOMM20 antibodies to determine the fusion of lysosomes and mitochondria, forming mitolysosomes. HIF‐1α downregulation increased LC3II and TOMM20 colocalization, suggesting HIF‐1α‐mediated suppression of mitophagy in the CRF cell model (Figure 5H,I). The effects of HIF‐1α on mitochondrial membrane potentials in the CRF cell model were assessed using TMRM uptake. Membrane potentials were found to be increased in HIF‐1α‐shRNA‐treated cells relative to the Model + sh‐CTRL group (*p* < 0.010; Figure 5J,K). Transmission electron microscopy (TEM) revealed disordered skeletal muscle fibre structure and a significant increase in mitophagosomes in the CRF groups. However, the number and appearance of mitochondria were markedly improved after downregulation of HIF‐1α in the CRF group (Figure 5L,M). Furthermore, we assessed the levels of the PINK1–Parkin pathway and found significant increases in the levels of PINK1 and Parkin in HIF‐1α‐shRNA‐treated cells compared to the Model + sh‐CTRL group (Figure 5N,O). Meanwhile, p62 expression was significantly inhibited in the Model + sh‐HIF‐1α group compared to the Model + sh‐CTRL group. These results indicate the critical role of HIF‐1α in mitophagy in the CRF cell model.

### 3.8 Influence of SFI on the levels of ROS and mitochondrial proteins in HIF‐1α‐overexpressing C2C12 cells cultured with CT26‐conditioned medium

**FIGURE 6:**
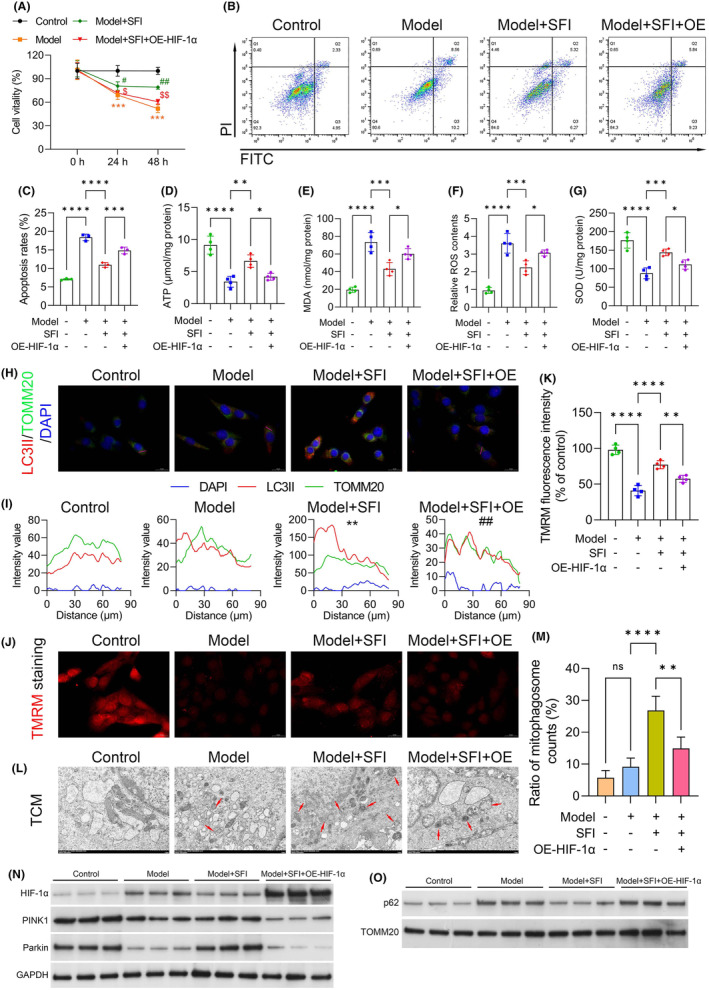
Effects of Shenqi Fuzheng injection (SFI) on the levels of reactive oxygen species (ROS) and proteins associated with mitochondrial function in HIF‐1α‐overexpressing cells cultured with CT26‐conditioned medium. (A) Cell viability shown by CCK‐8 assays. (B, C) Apoptosis assessed by flow cytometry. (D, E) Levels of adenosine triphosphate (ATP) and malondialdehyde (MDA). (F, G) Levels of ROS and superoxide dismutase (SOD). (H) Lysosome identification by microtubule associated protein 1 light chain 3 (LC3) II and translocase of outer mitochondrial membrane 20 (TOMM20) antibodies (Scale bar = 20 μm). (I) Colocalization analysis of LC3II and TOMM20. (J, K) TMRM uptake assay showing the effects of HIF‐1α on mitochondrial membrane potentials in the CRF model (Scale bar = 20 μm). (L, M) Electron micrographs showing the number and appearance of mitochondria (Scale bar = 1 μm). (N, O) Western blots showing the levels of proteins associated with the PTEN induced kinase 1 (PINK1)–Parkin pathway. ****p < 0.0001; ***p < 0.001; **p < 0.01; *p < 0.05; ns, nonsignificant.

We further investigated the cellular responses to HIF‐1α modulation in the context of SFI treatment. Cell viability and apoptosis were assessed in C2C12 cells following transfection with the HIF‐1α overexpression plasmid and treatment with SFI. HIF‐1α overexpression markedly increased cell viability following SFI treatment, indicating a reversal of the proliferation‐suppressing effects of SFI in C2C12 cells (Figure 6A). Moreover, the apoptosis‐suppressing effects of SFI were also counteracted by HIF‐1α overexpression, as evidenced by flow cytometry analysis (Figure 6B,C). Furthermore, we investigated the influence of HIF‐1α overexpression on mitochondrial function and oxidative stress in C2C12 cells treated with SFI. The results revealed that the MDA and relative ROS contents increased significantly following HIF‐1α overexpression plasmid transfection, while ATP and SOD levels were markedly reduced in the Model + SFI + HIF‐1α overexpression plasmid group relative to Model + SFI cells (Figure 6D–G). Additionally, the colocalization promotion effects of SFI on LC3II and TOMM20 were weakened by HIF‐1α overexpression (Figure 6H,I). Moreover, TMRM uptake assays demonstrated a significant decrease in the mitochondrial membrane potential in cells transfected with HIF‐1α overexpression plasmid and treated with SFI (Model + SFI + OE group) compared to the Model + SFI group (Figure 6J,K). Then, a TCM assay verified that the number and appearance of mitochondria were markedly impaired after upregulation of HIF‐1α in the SFI‐pretreated CRF group (Figure 6L,M). We also assessed the levels of the PINK1–Parkin pathway and found that the levels of PINK1 and Parkin were markedly reduced, while p62 was increased in the Model + SFI + OE group relative to the Model + SFI group (Figure 6N,O). These findings suggest that SFI may modulate ROS levels, mitophagy and mitochondrial function through the regulation of HIF‐1α via the PINK1–Parkin pathway.

### 3.9 HIF‐1α interacts with the PINK1 promoter and regulates its transcription

**FIGURE 7:**
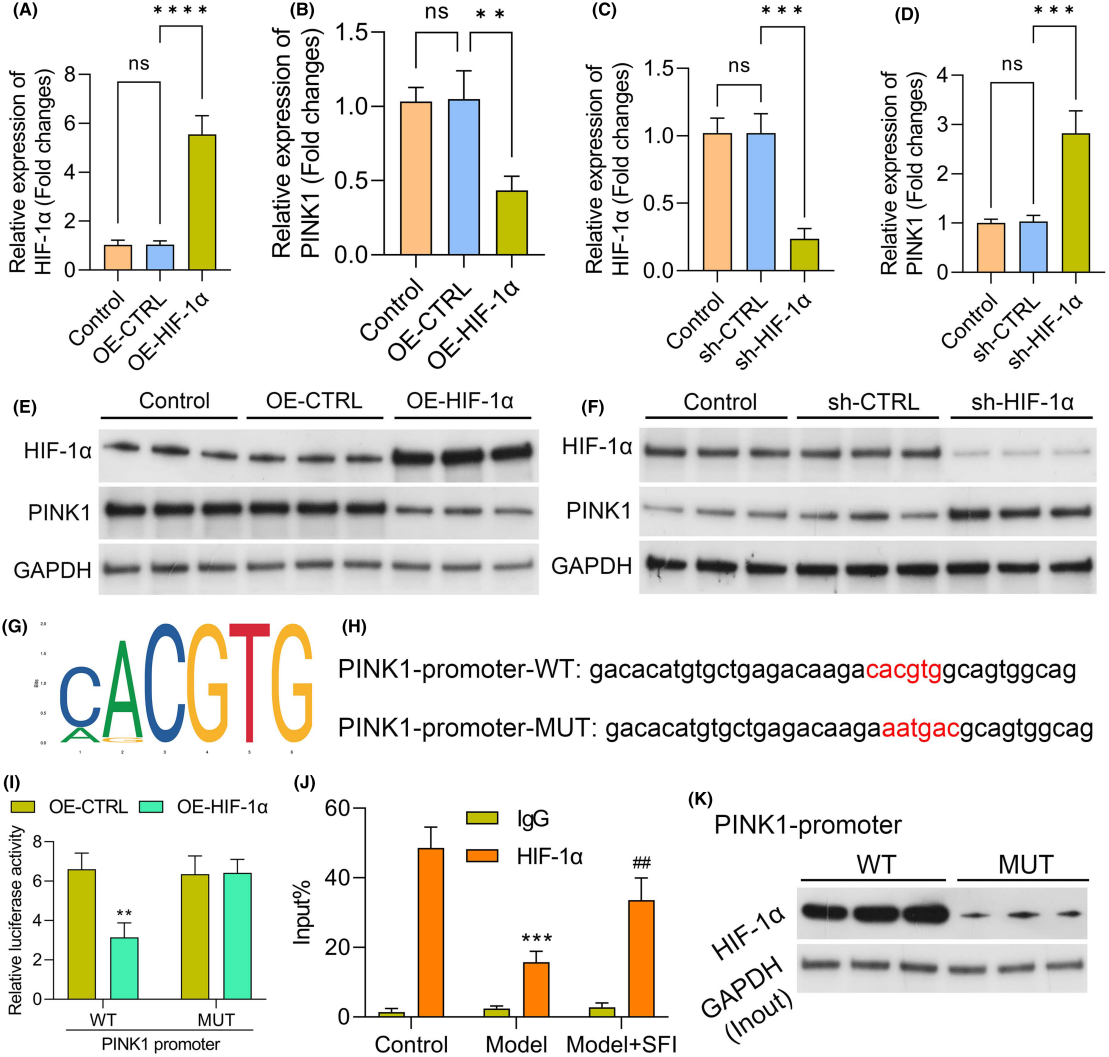
Hypoxia inducible factor 1 subunit alpha (HIF‐1α) binds directly to the PTEN induced kinase 1 (PINK1) promoter and modulates its transcription. Levels of HIF‐1α and PINK1 were verified by RT–qPCR or western blot in C2C12 cells after silencing or overexpressing HIF‐1α (A–F). Levels of PINK1 mRNA (B) and protein (E) in cells overexpressing HIF‐1α. Levels of PINK1 mRNA (D) and protein (F) after silencing of HIF‐1α. Results from the JASPAR database showing sequences of the HIF‐1α consensus binding sites. (G). Schematic representation of the sequences of the pGL3‐PINK1‐wild‐type and mutant plasmids (H). HIF‐1α‐overexpressing cells were transfected with pGL3‐PINK1‐wt or pGL3‐PINK1‐mut plasmids for 48 h. PINK1 promoter activity was determined by dual‐luciferase reporter gene assay (I). PINK1 levels after treatment with anti‐IgG or anti‐HIF‐1α antibodies, as shown by chromatin immunoprecipitation (ChIP) assays (J). Pulldown of HIF‐1α by biotinylated PINK1 oligos but not mutated oligos (K). ****p < 0.0001; ***p < 0.001; **p < 0.01; ns, nonsignificant.

In subsequent experiments, we delved into understanding the regulatory relationship between HIF‐1α and PINK1, specifically investigating whether HIF‐1α could modulate the influence of SFI on mitophagy through controlling PINK1 expression. Initially, we validated the altered expression of HIF‐1α and PINK1 in C2C12 cells after manipulating HIF‐1α levels through transfection with sh‐HIF‐1α and HIF‐1α overexpression plasmids, respectively. The upregulation of HIF‐1α in cells following transfection with the OE‐HIF‐1α plasmid and its downregulation after knockdown were confirmed via RT–qPCR and western blotting (Figure 7A–C,E,F). Importantly, we observed that HIF‐1α overexpression reduced PINK1 levels in C2C12 cells, while downregulation of HIF‐1α significantly increased PINK1 expression (Figure 7B,D,F). To explore the molecular basis of HIF‐1α regulation of PINK1, we utilized the JASPAR database to predict potential binding sequences of HIF‐1α within the PINK1 promoter region. A putative binding site was identified (Figure 7G). To validate the importance of this binding site, we performed dual‐luciferase assays. The results revealed that the sequence within the PINK1 promoter region was essential for HIF‐1α binding and the regulation of PINK1 promoter activity (Figure 7H,I). Additionally, ChIP analysis confirmed that anti‐HIF‐1α antibodies led to HIF‐1α protein enrichment in the regulatory region of the PINK1 gene when HIF‐1α was overexpressed (Figure 7J). Furthermore, we conducted pulldown assays using biotinylated PINK1 oligos. The results demonstrated the specific interaction of HIF‐1α with the PINK1 promoter region, as evidenced in the pulldown of HIF‐1α by the biotinylated PINK1 oligos but not by mutated oligos (Figure 7K). In summary, these findings provide compelling evidence supporting the direct regulation of PINK1 expression by HIF‐1α, shedding light on the intricate mechanisms through which HIF‐1α influences mitophagy, especially in the context of SFI treatment.

## 4 DISCUSSION

CRF remains a complex and challenging complication.^7^, ^9^ This study, integrating a comprehensive approach encompassing mitochondrial function, inflammation, immune response and mitophagy, sheds light on potential avenues for CRF intervention. When contextualized within the broader landscape of existing research, several critical points emerge.

Mitochondrial dysfunction, a key driver of CRF, underscores the therapeutic potential of targeting mitochondrial health, as evidenced by SFI's ability to mitigate these dysfunctions, offering a promising avenue for CRF management. Mitochondrial dysfunction has long been implicated in CRF. Cancer therapies, particularly chemotherapy and radiotherapy, disrupt mitochondrial homeostasis, leading to decreased ATP production and increased oxidative stress.^21^, ^22^, ^23^ The findings in this study corroborate existing research, emphasizing the centrality of mitochondrial health in CRF. Importantly, the ability of SFI to preserve mitochondrial structure and function aligns with emerging studies highlighting the significance of mitochondrial‐targeted therapies in managing cancer‐related symptoms.^24^, ^25^ The findings of this study not only reaffirm the centrality of mitochondrial health in CRF but also highlight the extraordinary potential of SFI in safeguarding the delicate balance of mitochondrial structure and function. In the intricate tapestry of CRF, these revelations herald a breakthrough, emphasizing the promise of targeted mitochondrial therapies in alleviating the burden of cancer‐related symptoms.

Inflammation and immune dysregulation are intertwined with CRF. The immunomodulatory effects of SFI, as demonstrated in this study, echo the findings in research exploring the role of the immune system in CRF.^26^, ^27^, ^28^ Chronic inflammation in cancer patients disrupts normal immune responses, contributing to fatigue. SFI's potential in modulating immune responses, possibly through the regulation of cytokines and immune cell activity, aligns with recent studies investigating immunotherapeutic strategies for CRF. This study paints a nuanced picture, revealing the intricate dance between immune responses and chronic inflammation, which both contribute significantly to the relentless onslaught of fatigue in cancer patients. The ability of SFI to modulate immune cell activity and potentially regulate cytokine profiles emerges as a beacon of hope, hinting at the possibility of restoring immune equilibrium in individuals grappling with CRF. As we delve deeper into the immunological underpinnings of CRF, SFI stands as a potential modulator, offering respite to patients beleaguered by the unrelenting impact of immune dysregulation.

The involvement of the HIF‐1α pathway in CRF, as elucidated in this study, presents a novel angle for exploration. HIF‐1α is a central player in cellular responses to hypoxia, inflammation and oxidative stress.^29^, ^30^ Dysregulation of HIF‐1α has been linked to mitochondrial dysfunction and impaired mitophagy, potentially exacerbating CRF.^31^ The ability of SFI to modulate HIF‐1α and enhance mitophagy underscores its potential in restoring mitochondrial health and cellular energy balance. This aligns with recent studies delving into the function of HIF‐1α in cancer‐related symptoms and the exploration of targeted therapies.^32^ Dysregulation of HIF‐1α has far‐reaching consequences, disrupting mitochondrial integrity and impairing mitophagy, potentially exacerbating CRF. Within this intricate molecular landscape, SFI emerges as a potential modulator, balancing the delicate interplay between HIF‐1α and mitochondrial function, offering a glimmer of hope to patients grappling with the debilitating effects of CRF.

Investigating specific pathways involved in SFI‐mediated HIF‐1α regulation and mitophagy enhancement could unravel precise therapeutic targets. Furthermore, exploring patient‐specific factors influencing SFI response, such as genetic variations or specific cancer mutations, could pave the way for personalized CRF management strategies. To date, when viewed in the context of existing research, these findings provide a robust foundation for further exploration. SFI's multifaceted effects on mitochondrial health, inflammation, immune responses and the HIF‐1α pathway highlight its potential as a promising intervention for CRF. Continued collaborative efforts between basic scientists, clinicians and pharmacologists are essential to harness the full therapeutic potential of SFI and improve the lives of cancer patients affected by CRF.

However, the specific regulatory mechanism of SFI in CRF mediated by HIF‐1α remains largely unknown. Thus, in this study, we investigated the molecular mechanisms underlying the effects of SFI on HIF‐1α and mitophagy. We confirmed that SFI could promote mitophagy with accelerated clearance of autophagosomes, thus increasing cell viability, reducing intracellular ROS levels and increasing mitochondrial membrane potential. In addition, this study further confirmed that the HIF‐1α/PINK1 pathway is a critical signalling pathway involved in SFI‐enhanced mitophagy. The expression levels of autophagy‐associated proteins and HIF‐1α signalling pathway proteins were quantified by western blot. We found that SFI could significantly upregulate the expression levels of the mitochondrial functional proteins PINK1 and Parkin and significantly downregulate the expression of the autophagy marker protein p62. We found that the protective effect of SFI was impaired when a HIF‐1α gene overexpression plasmid was used, suggesting that HIF‐1α is a key target protein involved in regulating mitophagy and that SFI promotes mitophagy against CRF through the HIF‐1α/PINK1 signalling pathway.

## 5 CONCLUSIONS

The present work demonstrated that SFI could promote mitophagy by suppressing HIF‐1α expression to remove dysfunctional mitochondria and alleviate CRF. These findings provide novel insights into the mechanisms of this new drug formulation for the future treatment of CRF. However, the underlying mechanisms are likely more complex than we described here, and our results do not exclude the possible involvement of other mechanisms caused by SFI to treat CRF.

## AUTHOR CONTRIBUTIONS

**Wei Guo:** Conceptualization (equal); data curation (equal); funding acquisition (equal); project administration (lead); visualization (equal); writing – original draft (lead); writing – review and editing (equal). **Shan Liu:** Data curation (lead); investigation (equal); supervision (equal); visualization (equal); writing – review and editing (equal). **Huan Xia:** Formal analysis (lead); investigation (equal); methodology (equal); validation (equal); writing – original draft (equal); writing – review and editing (equal). **Jiamin Luo:** Formal analysis (equal); writing – review and editing (equal). **Hanrui Chen:** Funding acquisition (equal); writing – review and editing (equal). **Leihao Hu:** Investigation (equal); writing – review and editing (equal). **Xinting Zheng:** Supervision (equal); writing – review and editing (equal). **Zhiwei Xiao:** Validation (equal); writing – review and editing (equal). **Lizhu Lin:** Conceptualization (lead); methodology (equal); project administration (equal); writing – review and editing (equal).

## FUNDING INFORMATION

This study was supported by the National Natural Science Foundation of China (82274598, 82304979), Department of Education of Guangdong Province (2023ZDZX2016), Guangdong Science and Technology Department (2021A1515011489), Characteristic Innovation Project of Ordinary Colleges and Universities in Guangdong Province (2020KTSCX028), Guangzhou Municipal Science and Technology Bureau (202002030376), the Guangdong Natural Science Foundation (2021A1515110785) and the Secondary Development Project of Famous Traditional Chinese Medicines in Guangdong Province.

## CONFLICT OF INTEREST STATEMENT

The authors declare that they have no competing interests.

## CONSENT

Not applicable.

## Supporting information


Data S1.

## Data Availability

The datasets used and/or analysed during the current study are available from the corresponding author on reasonable request.
