# Supplementary material for: Shenqi Fuzheng injection facilitates skeletal muscle mitophagy mediated by the ubiquitination of HIF‐1α to ameliorate cancer‐associated fatigue
Source: J Cell Mol Med. 2024 Jun 19;28(12):e18455. doi: 10.1111/jcmm.18455 (PMC11187406; doi:10.1111/jcmm.18455)
Supplement: Supplementary file 1 — Data S1. [file JCMM-28-e18455-s001.docx]

**Supplementary materials**

**Materials and Methods**

**Cells and culture**

The C2C12 and CT26 cell lines were procured from the Cell Bank of the Chinese Academy of Sciences (Shanghai, China). Cells were grown in DMEM (Sigma‒Aldrich, St. Louis, MO, USA) with 10% fetal bovine serum (FBS; Gibco, Waltham, MA, USA) at 37 ℃ and 5% CO_2_. C2C12 cells (3×10^4^/well) were inoculated in 96- or 6-well plates (5 × 10^5^/well) and grown for 24 h. Media were then substituted with DMEM supplemented with 2% horse serum (Gibco) and grown for three days for myotube induction. All experiments were performed with mycoplasma-free cells.

In our experiments, we manipulated HIF-1α expression in C2C12 cells using two distinct molecular techniques. For knockdown, specific shRNA sequences targeting HIF-1α were designed and synthesized. These shRNA sequences were cloned and inserted into a suitable vector and transfected into C2C12 cells using Lipofectamine 2000, leading to a reduction in HIF-1α expression. For overexpression, a plasmid carrying the HIF-1α gene was constructed and transfected into C2C12 cells, resulting in elevated levels of HIF-1α. Transfected cells were selected using appropriate antibiotics, and the efficacy of knockdown or overexpression was validated through techniques such as RT-PCR and Western blotting. These methods allowed us to establish C2C12 cell models with either reduced or enhanced HIF-1α expression, facilitating the exploration of its functional implications in our study.

**SFI treatment in C2C12 myotubes**

Mouse C2C12 myoblasts (ATCC) were cultured in proliferation medium (DMEM + 10% FBS, 1% P/S) until 70% confluency was reached. Differentiation was induced in DMEM with 2% horse serum and 1% P/S, and the medium was changed every 2 days. Fully differentiated myotubes were treated with CT26 culture medium (CM) to create a CRF cell model. Groups included a Control (no treatment), a Model group (CM treatment), and an SFI group (CM with 5 mg/mL SFI).

**CCK-8 assay**

The CCK-8 detection kit was sourced from Bioss (BA00208, China). The viability of C2C12 cells was evaluated by CCK-8 assays according to the provided protocol. Cells (5 × 10³/well) in logarithmic growth phase were inoculated in 96-well plates and grown for 24 and 48 h, after which 20 μL/well of CCK-8 reagent was added, and absorbances at 450 nm were read in a microplate reader. Quadruplicate samples were tested for each concentration.

**Detection of apoptosis in C2C12 myotubes**

Apoptosis in myotubes 48 hours posttreatment was assessed using an Annexin V-FITC/PI Kit. Myotubes were collected, suspended in Annexin V binding buffer, and incubated with AV-FITC and PI reagents for 20 minutes at 37 ℃. The cells were analyzed by flow cytometry on a FACSCanto instrument.

**RNA extraction and real-time qPCR analysis**

Total RNA was extracted using TRIzol reagent following the manufacturer’s protocol. Subsequently, reverse transcription was carried out using a qPCRBIO cDNA synthesis kit and amplified by RT‒qPCR using the qPCRBIO fast qPCR SyGreen blue mix. The following primers were used: HIF-1α forward primer: 5′-CTTGACAAGCTAGCCGGAGG-3′, reverse primer: 5′-AAGAGACAAGTCCAGAGGCG-3′; Pink1 forward primer: 5′-ATCTGGTTCAGCAGGGCATT-3′, reverse primer: 5′-AGGGACAGCCATCTGAGTCC-3′; GAPDH forward primer: 5′-GGAGAGTGTTTCCTCGTCCC-3′, reverse primer: 5′-ATGAAGGGGTCGTTGATGGC-3′. The quantification of HIF-1α transcript expression levels was performed relative to GAPDH and computed utilizing the 2^-ΔΔCt^ method, where ΔΔCt indicates the difference in threshold cycles between the target gene (HIF-1α) and the internal control (GAPDH). This method enabled a precise assessment of HIF-1α gene expression levels, ensuring accurate comparisons and reliable interpretation of the results.

**Mitophagy Assessment**

Mitophagy was evaluated using a combination of immunostaining for LC3II and TOMM20, transmission electron microscopy (TEM), and analysis of mitochondrial membrane potential.

**Mitochondrial function**

ATP levels were quantified in lysates derived from 10^5^ cells utilizing the ATP Bioluminescence Assay Kit, according to the provided directions. This method harnesses the ATP dependence of luciferase-catalyzed luciferin oxidation, which emits light in proportion to the ATP concentration. By measuring this luminescence, the cellular ATP content was accurately determined, providing valuable insights into the cellular energy status.

**ROS and MDA Measurement**

ROS levels were quantified using the 2',7'-dichlorofluorescin diacetate (DCFDA) cellular ROS detection assay according to the manufacturer's protocol. Fluorescence intensity was measured using a fluorescence microplate reader. Malondialdehyde (MDA) levels, an indicator of lipid peroxidation, were determined using the MDA assay kit following the manufacturer's instructions. The absorbance was measured spectrophotometrically.

**Immunofluorescence**

In the immunofluorescence staining procedure, C2C12 cells were colabeled using antibodies against LC3 and TOMM20, key markers for autophagosomes and mitochondria, respectively. Cells were fixed with 4% paraformaldehyde, permeabilized with 0.1% Triton X-100, and blocked with 5% BSA., the cells were then incubated with primary antibodies against LC3 (ab192890, Abcam, Cambridge, UK) and TOMM20 (ab186735, Abcam, Cambridge, UK) overnight at 4 °C. After thorough washing, the cells were then exposed to corresponding secondary antibodies conjugated with fluorescent dyes. Subsequently, confocal microscopy was used for visualization. Analysis of LC3 and TOMM20 signal colocalization indicated the association between autophagosomes and mitochondria in C2C12 cells. This immunofluorescence staining technique allowed for the precise detection and visualization of the interactions between LC3 and TOMM20, providing essential insights into cellular processes and organelle dynamics in C2C12 cells.

**Mitochondrial Membrane Potential Assessment**

To investigate mitochondrial membrane potential, we utilized tetramethylrhodamine ethyl ester (TMRM) staining, a cell-permeant, red‒orange fluorescent dye that selectively accumulates in active mitochondria. A TMRM kit for mitochondrial membrane potential assessment was obtained from Beyotime (Shanghai, China). Concurrently, MitoTracker Green staining was applied to quantify mitochondrial mass, irrespective of membrane potential. This dual staining approach provided comprehensive evaluations of both mitochondrial activity and mass within the cellular context, offering valuable insights into mitochondrial function.

**Mouse model**

The study protocol was devised prior to commencement of the study without formal registration. Animal experiments adhered to the guidelines outlined by The First Affiliated Hospital of Guangzhou University of Chinese Medicine (No. 20210827003). BALB/C nude mice (female, six weeks old, 20–22 g) were housed under controlled conditions of temperature, humidity, and light-dark cycles. There were no specific criteria for animal inclusion or exclusion, and humane endpoints were not predefined in this study. In summary, 12 BALB/c mice received intraperitoneal injections of CT26 cells (1×10^6^/100 µL PBS) and were randomly allocated to two groups, namely, the model and SFI groups, with six mice per group. Randomization was carried out using computer-generated numbers with a 1:1:1 ratio in sealed opaque envelopes. The Model group was treated with PBS by oral gavage, while those in the SFI group received SFI (3 g/kg/day), with the dose determined through preexperimentation. The SFI was prepared in the pharmacy department of the hospital. The Control group (n = 6) received intraperitoneal injections of 0.1 mL PBS and oral gavage of an equal volume of PBS. Oral administration was conducted daily for 15 days, and the mice were weighed on alternate days, with body weight measurements taken every 2 days. Behavioral assessments were conducted before euthanasia, as previously described ^1^.

**Ethology assessment**

In the exhaustive swimming test (EST), lead pendants, representing 7% of the body weight, were attached to the tails of the mice before and on Days 10 and 20 postadministration. The animals were then placed in a container 30 cm deep and 25 cm in diameter filled with water at 25 °C ± 1 °C. The duration the mice could swim before exhaustion, noted when their noses were submerged for a minimum of 10 seconds, was recorded.

In the open-field test (OFT), we employed the Chengdu Tai League^TM^ visual behavioral assessment system. The mice were placed in the center of a box, and their subsequent movements, including total distance moved and time spent immobile, were recorded for 5 minutes. The box was cleaned meticulously before each mouse was tested.

In the tail suspension test (TST), following the predetermined groupings and administration plans, all mice from the OFT were assessed. Thirty minutes after the final administration, the mouse’s tail (2 cm from the tip) was affixed to a TS-200 tail-suspension tester, with the head of the mouse situated 5 cm above the base of the tester. The time each mouse remained immobile was monitored for 5 minutes.

**Hematoxylin and eosin staining**

Samples of the gastrocnemius muscle were fixed (4% paraformaldehyde), embedded, and stained with hematoxylin and eosin (HE) using a kit from Servicebio Technology Co., Ltd. (Wuhan, China). Myofiber cross-sectional areas were evaluated. Additional tissue specimens were fixed in glutaraldehyde buffer (Solarbio Life Science and Technology, Beijing, China) for 6 h at 4 ℃, followed by dehydration, embedding, and sectioning. The sections were stained with both lead citrate and uranyl acetate (HEAD Biotechnology Co., Ltd., Beijing, China).

**Electron microscopy**

For tissues, gastrocnemius muscle samples and treated cells were prepared for electron microscopy analysis. After fixation with 2.5% glutaraldehyde, postfixation in 1% osmium tetroxide, dehydration in an ethanol gradient, and embedding in epoxy resin, ultrathin sections were cut with an ultramicrotome. The sections were contrast-stained with lead citrate and uranyl acetate and examined under transmission electron microscopy. This process allowed for the analysis of cellular ultrastructure and mitochondrial health in the gastrocnemius muscle across different experimental groups. For cells, Cells were fixed with 2.5% glutaraldehyde, post-fixed with 1% osmium tetroxide, dehydrated in graded ethanol, and embedded in epoxy resin. Ultrathin sections were stained with uranyl acetate and lead citrate and examined under a transmission electron microscopy (TEM).

**Measurement of mtDNA**

DNA extraction was performed using an AccuPrep® Genomic DNA extraction kit (Bioneer Corporation) according to the manufacturer’s directions. The levels of mitochondrial DNA (mtDNA) were determined by real-time PCR. This method, targeting the mitochondrial 16S rRNA gene, is a widely recognized protocol for measuring mitochondrial contents. The primers used have been previously described^2^.

**Chemiluminescence assay for MDA and SOD detection**

In our study, we utilized a chemiluminescence assay to quantify malondialdehyde (MDA) levels and assess superoxide dismutase (SOD) activity in both C2C12 cells and mouse skeletal muscle tissues. The reactive oxygen species assay kit was acquired from Solarbio Science & Technology Co., Ltd. (Beijing, China). For C2C12 cells, harvested samples were lysed, and cellular components were extracted. In the case of mouse skeletal muscle tissues, homogenization was performed to obtain the necessary components. A chemiluminescent MDA assay kit was employed to measure MDA levels, indicating lipid peroxidation, while SOD activity was assessed using a chemiluminescent SOD assay.

**Biochemical indices**

Blood was collected from the CRF model mice and centrifuged (2,500 rpm, 20 min) to obtain the serum. The levels of hemoglobin (Hb), hematocrit (HCT), aspartate aminotransferase (AST), alanine aminotransferase (ALT), total protein (TP), globulin (GLOB), albumin (ALB), creatinine (CR), and blood urea nitrogen (BUN) were measured using either a hemoglobin analyzer (URIT, China) or a fully automatic animal biochemistry analyzer (URIT). The levels of adenosine triphosphate (ATP), superoxide dismutase (SOD), and malondialdehyde (MDA) were determined in gastrocnemius muscle samples using kits (Jiancheng Medical Technology Co., Ltd., Jiancheng, China) following the respective directions.

**Sample preparation and iTRAQ labeling**

Gastrocnemius muscle samples from the different groups (SFI-treated Model, Model, and Control groups) were pooled in equal volumes for all 18 subjects within the respective groups. High-abundance proteins were discarded using a Human 14 Multiple Affinity Removal System (Agilent, Santa Clara, CA, USA). The extracted proteins were quantified using a Bradford Protein Assay Kit (Bio-Rad, Hercules, CA, USA). One hundred milligrams of protein from each sample was reduced and alkylated, followed by enzymatic hydrolysis with trypsin. Following digestion, the peptides were labeled using an 8-plex iTRAQ reagent kit (Applied ABI, USA) according to the manufacturer’s directions. After labeling, the three samples were combined, desalted on a Sep-Pak Vac C18 column (Waters, USA), and lyophilized.

**Western blot analysis**

Following RIPA buffer-mediated protein extraction and quantification through the use of a BCA assay (Beyotime, Shanghai, China), proteins were separated through electrophoresis prior to their transfer onto PVDF membranes (Millipore, MA, USA). Blots were blocked with 5% skim milk for 2 h, probed with primary and secondary antibodies, imaged via enhanced chemiluminescence (ECL), and analyzed using ImageJ (NIH, MD, USA). Primary antibodies included anti-HIF-1α (1:1000, ab179483, Abcam, Cambridge, UK), anti-Pink 1 (1:1000, abs117684, Absin, Shanghai, China), anti-p62 (1:1000, ab109012, Abcam, Cambridge, UK), anti-Parkin (1:1000, ab77924, Abcam, Cambridge, UK), anti-TOMM20 (1:1000, ab186735, Abcam, Cambridge, UK), and anti-GAPDH (1:2000, ab8245, Abcam, Cambridge, UK).

**Coimmunoprecipitation (co-IP) assay**

In the immunoprecipitation procedure, whole-cell lysates were incubated with appropriate antibodies overnight, facilitating the formation of antibody-protein complexes. After incubation, the lysate-antibody complexes were incubated with protein A/G beads (2‒4 h, 4 °C). Following incubation, the beads were subjected to thorough washing (3 times) with NP-40 lysis buffer to eliminate nonspecific binding. Elution of the immunoprecipitated proteins from the beads was performed by mixing them with 2×SDS sample buffer, followed by boiling for 5-10 minutes to denature the proteins. The resulting eluate, containing the coprecipitated proteins and antibodies, was then subjected to analysis via Western blotting techniques.

**Dual-luciferase assay**

The JASPER database was used to predict the interaction site between HIF-1α and the promoter region of human PINK1. The wild-type and mutant PINK1 promoters were inserted into the luciferase reporter vector pGL4.0. Recombinant plasmids were cotransfected with plasmid pRL-TK into C2C12 cells, and the luciferase activities were measured by a dual-luciferase reporter system. The firefly and Renilla luciferase activity levels were detected sequentially from a single sample using a DLR assay kit (Promega, Madison, WI, USA). The results were analyzed with reference to the Renilla luciferase activity. All experiments were carried out in triplicate, and three independent experiments were performed.

**Chromatin immunoprecipitation assay**

Chromatin immunoprecipitation assays were performed to explore the interaction between HIF-1α and the promoter region of the PINK1 gene. Cells were cross-linked using 1% formaldehyde for 10 minutes at room temperature to preserve protein-DNA interactions. The cross-linking reaction was quenched with 125 mM glycine. Chromatin was then sheared to an average length of 200–1000 bp using a sonicator. Sheared chromatin was pre-cleared with protein A/G magnetic beads and subsequently incubated overnight at 4°C with 5 µg of anti-HIF-1α antibody or normal rabbit IgG as a negative control. Protein-DNA complexes were captured using protein A/G magnetic beads, washed, and eluted from the beads. Cross-links were reversed by overnight incubation at 65 °C with sodium chloride. DNA was then purified using a DNA purification kit. For the quantitative analysis of ChIP samples, PCR amplification was conducted using specific primers targeting the regulatory region of the PINK1 gene. The primer sequences used for amplifying the PINK1 promoter region were as follows: Forward Primer: 5’-CAGCTGAGACAGACACATGT-3’ and Reverse Primer: 5’-GTCCTATTTATATTCCCTCC-3’. The PCR conditions were as follows: initial denaturation at 95°C for 3 minutes, followed by 35 cycles of 95°C for 30 seconds, 58°C for 30 seconds, and 72°C for 30 seconds, with a final extension at 72°C for 5 minutes. PCR products were analyzed on a 2% agarose gel to confirm the specificity of the amplification. Quantitative PCR (qPCR) was also performed to quantify the enrichment of HIF-1α binding to the PINK1 promoter region relative to the input chromatin. Data were normalized to the IgG control to account for nonspecific binding.

**DNA Pulldown**

The biotinylated PINK1 promoter probe, essential for studying gene regulation, was meticulously synthesized by Sangon (Shanghai, China). In the experimental procedures, 400 μg of nucleoprotein extract was carefully combined with 4 μg of the PINK1 promoter probe and streptavidin-agarose beads (45 μl, Sigma). This amalgamation was incubated at ambient room temperature, allowing the formation of DNA‒protein complexes. After a carefully monitored incubation period of 2 hours, the resulting complex, representing a critical snapshot of intricate molecular interactions, was carefully collected through centrifugation.

**Reference:**

1. Zhang S, Gong F, Liu J, Liu T, Yang J, Hu J. A novel PHD2 inhibitor acteoside from Cistanche tubulosa induces skeletal muscle mitophagy to improve cancer-related fatigue. *Biomed Pharmacother*. Jun 2022;150:113004. <https://doi.org10.1016/j.biopha.2022.113004>.

2. Yuan Y, Zheng Y, Zhang X, et al. BNIP3L/NIX-mediated mitophagy protects against ischemic brain injury independent of PARK2. *Autophagy*. Oct 3 2017;13(10):1754-1766. <https://doi.org10.1080/15548627.2017.1357792>.
